# Space Use and Habitat Selection by Resident and Transient Coyotes (*Canis latrans*)

**DOI:** 10.1371/journal.pone.0132203

**Published:** 2015-07-06

**Authors:** Joseph W. Hinton, Frank T. van Manen, Michael J. Chamberlain

**Affiliations:** 1 Warnell School of Forestry and Natural Resources, University of Georgia, Athens, Georgia, United States of America; 2 U.S. Geological Survey, Northern Rocky Mountain Science Center, Interagency Grizzly Bear Study Team, Bozeman, Montana, United States of America; University of Sassari, ITALY

## Abstract

Little information exists on coyote (*Canis latrans*) space use and habitat selection in the southeastern United States and most studies conducted in the Southeast have been carried out within small study areas (e.g., ≤1,000 km^2^). Therefore, studying the placement, size, and habitat composition of coyote home ranges over broad geographic areas could provide relevant insights regarding how coyote populations adjust to regionally varying ecological conditions. Despite an increasing number of studies of coyote ecology, few studies have assessed the role of transiency as a life-history strategy among coyotes. During 2009–2011, we used GPS radio-telemetry to study coyote space use and habitat selection on the Albemarle Peninsula of northeastern North Carolina. We quantified space use and 2^nd^- and 3^rd^-order habitat selection for resident and transient coyotes to describe space use patterns in a predominantly agricultural landscape. The upper limit of coyote home-range size was approximately 47 km^2^ and coyotes exhibiting shifting patterns of space use of areas >65 km^2^ were transients. Transients exhibited localized space use patterns for short durations prior to establishing home ranges, which we defined as “biding” areas. Resident and transient coyotes demonstrated similar habitat selection, notably selection of agricultural over forested habitats. However, transients exhibited stronger selection for roads than resident coyotes. Although transient coyotes are less likely to contribute reproductively to their population, transiency may be an important life history trait that facilitates metapopulation dynamics through dispersal and the eventual replacement of breeding residents lost to mortality.

## Introduction

Similar to other *Canis* species, coyotes establish and hold territories to ensure optimal reproductive fitness through group living [1–4]. However, not all coyotes defend territories and biologists studying coyote ecology often classify them according to their space use as residents and transients [5–8]. Resident coyotes are individuals (breeders, juveniles, and pups) belonging to a pack and in possession of a territory that exhibit passive (i.e., scent marking) and aggressive (i.e., physical conflict) behaviors to exclude conspecifics [9,10]. Conversely, transient coyotes do not maintain territories and exhibit nomadic movements with no fidelity for any one area [5,8]. Researchers have traditionally focused on resident animals when studying space use of coyotes because residents make up the breeding portion of populations. Until recently, residents have been easier to study because their site fidelity and predictable movement patterns favor traditional telemetry techniques (i.e., very high frequency [VHF]) that require intensive field effort to locate study animals. Conversely, space use by transient coyotes has rarely been assessed because transients traverse expansive areas and are difficult to track without global positioning system (GPS) and satellite technology. A number of studies have noted the presence of coyotes with nomadic behaviors that traverse expansive areas and are difficult to monitor via VHF radio-telemetry [6,11–13]. For example, Andelt [6] reported that coyotes considered to be transients in his study were located <50% of the time within their study area and Chamberlain et al. [11] reported 33% of coyotes with VHF radio collars had permanently left their study area. Despite these logistical challenges, several studies have documented and assessed space use patterns of transient coyotes [6,7,14], but fewer have assessed both space use and habitat selection [8,15].

Coyote space use has been routinely studied and study area sizes ranged from approximately 30 km^2^ [6] to approximately 3,000 km^2^ [16]. Many well-referenced studies have been conducted within study areas about 1,000 km^2^ or less [6–8,17–19]. Because coyotes are highly mobile, patterns of space use and habitat selection within relatively small study areas can only provide part of the total knowledge into the spatial ecology of coyotes. Recently, Hinton et al. [20] described unique, localized space use during long-distance movements by 3 transient coyotes. They referred to intermittent, localized space use exhibited by transients as “biding” areas because those patterns may represent attempts by transients to assess areas and establish home ranges. Although Hinton et al. [20] reported anecdotal findings, their study indicated that assessing transient space use and habitat selection over broad geographic areas may provide important insights into how coyotes seek out and acquire territories.

Previous studies examining space use and habitat selection of coyotes concluded that transients are likely subordinate individuals who may actively avoid territories of residents and occupy suboptimal habitats not used by residents [5,7,8,15]. Additionally, Camenzind [5] suggested that transients serve as a surplus of individuals that are periodically recruited into the resident, reproductive segment of the population. These insights demonstrate that space and reproductive opportunities are limiting resources for coyotes. However, the ephemeral nature of space use that results from continuous exchanges of territorial ownership among individuals in coyote populations has been difficult to assess. Understanding these spatiotemporal dynamics is particularly important because they may contribute to life history characteristics of coyotes that permit populations to expand and persist in human-altered landscapes.

Extensive movements by transients involve decisions by individuals that contribute to key aspects of coyote ecology such as competition, foraging behavior, and habitat selection, which, in turn, influence population structure and processes over broad geographic areas. Because estimates of density, dispersal, and survival may be biased within small study areas [21,22], we define a minimum geographic extent as ≥2,500 km^2^. In the eastern United States, this large extent is important to capture actual dispersal ability of large *Canis* species and thus for proper classification of coyote social status [20,23]_._ Coyotes in eastern North Carolina are sympatric with endangered red wolves (*Canis rufus*) and both species are managed and monitored by the United States Fish and Wildlife Service (USFWS) Red Wolf Recovery Program (Recovery Program) on the Albemarle Peninsula to prevent hybridization and facilitate red wolf recovery [24,25]. Because Recovery Program biologists radio monitor both coyotes and red wolves throughout the Albermarle Peninsula, the approximately 6,000 km^2^ Red Wolf Recovery Area offers a large study area in the Southeast to evaluate the ecology of resident and transient coyotes.

Our understanding of key traits that facilitate coyote adaptation to diverse ecosystems throughout North America remains incomplete because studies examining the ecology of transient coyotes are limited. Understanding how coyote populations structure themselves on the landscape and which landscape characteristics facilitate coyote movements is critical for making reliable inferences about coyote ecology. Here, we compare space use and habitat selection by resident and transient coyotes to describe how coyotes exploit space. Our first objective was to quantify the size of areas used by resident and transient coyotes and describe the habitat composition of those areas. Our second objective was to assess differences in resident and transient habitat selection and develop resource-selection functions (RSFs) to map relative probability of habitat use by coyotes within the Recovery Area.

## Materials and Methods

**Fig 1:**
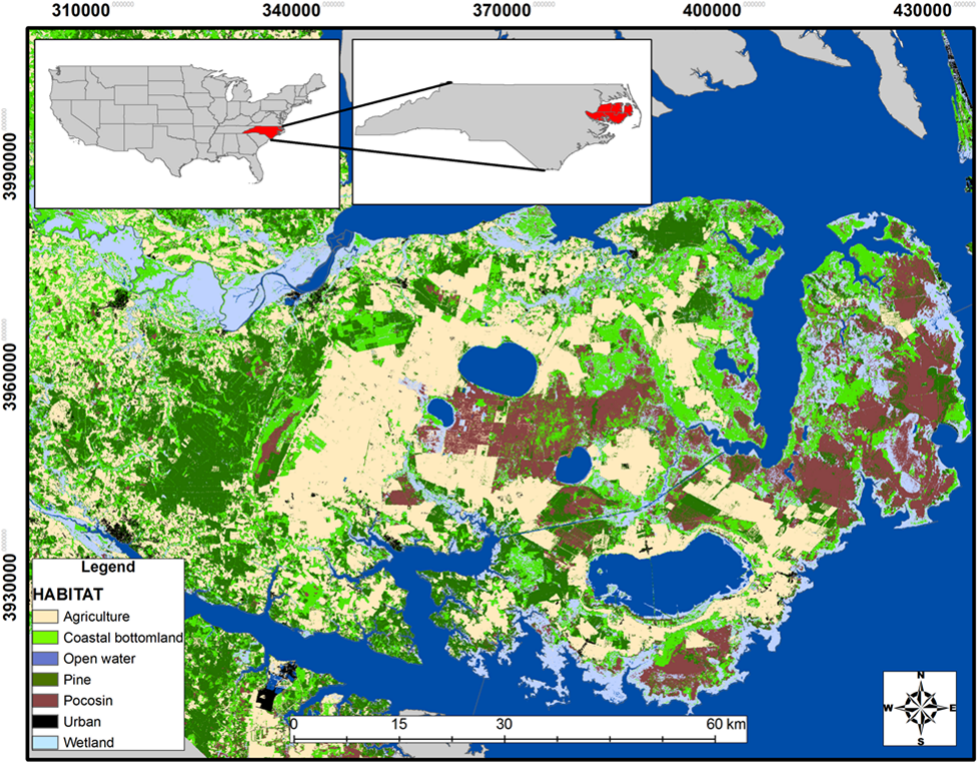
Map of the Albemarle Peninsula of northeastern North Carolina with primary habitat types during 2009–2011.

Our study was conducted on the Albemarle Peninsula in the northeastern region of North Carolina (Fig 1). The study area included approximately 6,000 km^2^ of federal, state, and private lands comprising a row-crop agricultural-bottomland forest matrix with little change in elevation (<50 m). Agricultural crops (i.e., corn, cotton, soybean, and winter wheat) and managed pine (*Pinus* spp.) composed of approximately 30% and 15% of the land cover, respectively. Other prominent land-cover types were coastal bottomland forests and pocosin (peatlands with a low [1–4 m] and dense evergreen shrub layer; 35%), herbaceous wetlands and saltwater marshes (5%), open water (5%), and other minor land-cover types (10%). The climate was typical of the mid-Atlantic: 4 distinct seasons, nearly equal in length, with an annual precipitation averaging between 122 to 132 cm. Summer climate was typically hot and humid with daily temperatures ranging from 27°C to over 38°C and winters were relatively cool with daily temperatures ranging between -4° to 7° C.

As part of long-term monitoring and management of red wolves and coyotes on the Albemarle Peninsula, the Recovery Program conducted annual trapping during autumn and winter to capture and fit individual red wolves and coyotes with radio collars. Our field study assisted annual trapping efforts from 2009 through 2011 to capture coyotes and red wolves. Coyotes were not a listed or protected species and the permitting authority for their capture and release was the North Carolina Wildlife Resources Commission. However, red wolves were listed as critically endangered by the International Union Conservation of Nature’s (IUCN) red list of threatened species and we operated under a cooperative agreement with the USFWS that permitted us to trap under special handling permits issued to the Recovery Program to trap and handle red wolves. This study, including all animal handling methods, was approved by the Louisiana State University Agricultural Center Institutional Animal Care and Use Committee (Protocol Number AE2009-19) and meets the guidelines recommended by the American Society of Mammologists [26]. Permission to access private lands for trapping occurred under memorandum of agreements (MOAs) between individual landowners and the Recovery Program. We access private lands of landowners without existing MOAs by contacting those individuals to receive permission to trap their lands.

We captured coyotes using padded foot-hold traps (Victor no.3 Softcatch, Woodstream Corporation, Lititz, Pennsylvania, USA) from October through May, 2009–2011. Coyotes were typically restrained using a catchpole, muzzle, and hobbles. Although most coyotes were not anesthetized, several were chemically immobilized with an intramuscular injection of ketamine HCl and xylazine HCl to inspect inside the mouth for injuries. Coyotes were sexed, measured, weighed, and aged by tooth wear [27], and a blood sample was collected. We categorized coyotes >2 years old as adults, 1–2 years old as juveniles, and <1 year old as pups. Coyotes on the Albemarle Peninsula were reproductively sterilized by the USFWS to prevent introgression into the red wolf population [24,25]. Coyotes were taken to a local veterinary clinic for surgical sterilization where males and females were reproductively sterilized by vasectomy and tubal ligation, respectively. This process keeps hormonal systems intact to avoid disrupting breeding and territorial behavior [28,29]. Prior to release at the original capture sites, we fit coyotes with a mortality-sensitive GPS radio collar (Lotek 3300s, Newmarket, Ontario, Canada) scheduled to record a location every 4 hours (0:00, 04:00, 08:00, and so on) throughout the year.

The Recovery Program monitored radio-collared red wolves and coyotes 2 times a week from aircraft to identify red wolf and coyote territories on the Albemarle Peninsula. Resident pairs of coyotes were identified as radio-collared individuals of breeding age (≥2 years old) who were temporally and spatially associated with one another and defending a territory for ≥4 months. When trapping was not feasible after radio-collared coyotes established territories, we confirmed the presence of a mate via field inspection for sign (i.e., visual observations and tracks) of another individual over the course of several weeks. To avoid autocorrelation, we only fit one coyote in each pair of residents with a GPS radio-collar. We classified radio-collared coyotes as transients when they were solitary and not associated with other radio-collared coyotes and displayed extensive movements throughout the Albemarle Peninsula.

To reflect the anthropogenic effects of agricultural practices on the landscape, we divided each year into 2 6-month seasons based on agricultural activity: growing (1 March–31 August) and harvest (1 September–28 February). We estimated space use of resident and transient coyotes by fitting dynamic Brownian bridge movement models (dBBMMs) to the time-specific location data to estimate the probability of use along the full movement track of each coyote [30], using R package moveud [31] in Program R [32]. Brownian bridge movement models use characteristics of an animal’s movement path among successive locations to develop a utilization distribution of an animal’s range. Because many factors influence telemetry error and recent studies suggest telemetry error for GPS radio collars range between 10–30 m [33], we used an error estimate of 20 m for all locations. Our error estimate was calculated based on recommendations and assumptions outlined in Byrne et al. [34]; we chose a moving window size of 7 locations (equivalent to 14 hours) with a margin of 3 locations for full tracks of each animal to reflect temporal shifts in coyote movements related to photoperiods. For residents, we considered 95% and 50% contour intervals as home ranges and core areas, respectively. Because transients do not maintain and defend territories, we did not refer to transient space use as home ranges and core areas. Instead, we considered 95% and 50% contour intervals for transients as transient ranges and biding areas [20], respectively. We used *t*-tests to investigate changes in the area of space use among seasons.

We estimated predominant landscape features from a digitized landscape map of vegetative communities developed by the North Carolina Gap Analysis Project [35]. We collapsed vegetative communities estimated by McKerrow et al. [35] into 4 general habitat classes with a 30-m resolution. For the habitat selection analysis, we divided the landscape into agriculture, coastal bottomland forest, pine forest, and wetlands (e.g., herbaceous wetlands, marshes, and pocosin). Because coyotes are known to use roads and forage along edges, we also developed road and agricultural-forest edge layers [36]. We created distance raster maps for habitat classes, roads, and agricultural-forest edges (hereafter edges) using the ‘Euclidean Distance’ tool in the Spatial Analyst toolbox in (ArcGIS 10; Environmental Systems Research Institute Inc., Redlands, California) to calculate the distance from every 30 m pixel to the closest landscape feature [37, 38]. We used analysis of variance (ANOVA) and Tukey tests [39] for multiple comparisons to determine if habitat composition of home ranges, core areas, transient ranges, and biding areas differed.

We used RSFs to examine relationships between landscape features and coyote establishment of home ranges on the landscape (2^nd^-order selection) [40] and to examine relationships between landscape features and coyote use within their home ranges (3^rd^-order selection) following Design II and III approaches suggested by Manly et al. [41]. For 2^nd^-order selection, we used individual animals as our sampling units and measured resource availability at the population level. For 3^rd^-order selection, we used individual animals as our sampling units and resource availability was measured for each animal. Despite the presence of territorial red wolves on the Albemarle Peninsula and active management by the Recovery Program to reduce red wolf-coyote hybridization, coyotes were found throughout the entire peninsula. We used distance-based variables to assess habitat selection to eliminate the need to base inference on subjectively chosen reference categories [37]. Therefore, we inferred “selection” when known (used) locations were closer to resource features than were random (available) locations and “avoidance” was inferred when known locations were farther from resource features than random locations. We used a binomial approach to estimate resource-selection functions by comparing characteristics of known locations to an equal number of random locations within the Albemarle Peninsula study area (2^nd^-order selection) and within home ranges and transient ranges (3^rd^-order selection) of coyotes [41]. We used generalized linear mixed models with a logistic link to compare habitat selection between resident and transient coyotes. We included random intercepts for individual coyotes in each model to account for correlation of habitat use within individuals and the unbalanced telemetry data. We modeled resource selection using the R package ‘lme4’ [42] with a binary (0 = available, 1 = used) response variable. Prior to modeling, we rescaled values for all distance-based variables by subtracting their mean and dividing by 2 standard deviations [38,43].

We designed 5 candidate models for coyote occurrence guided by 4 *a priori* general hypotheses to develop RSFs: (1) Coyotes require cover and shelter found primarily in forests. (2) Coyotes favor linear landscape characteristics, such as edges and roads. (3) Coyotes prefer open, treeless habitats, such as agricultural fields. (4) Coyotes avoid wetland habitats. We used an information-theoretic approach to assess models by calculating Akaike’s information criterion for small sample sizes (AIC_c_) [44,45] and used ΔAIC_c_ to select which models best supported habitat selection. First, we used all resident and transient locations from our telemetry data, included main effects for all fixed predictor variables, and considered interactions between a coyote status variable (resident = 1, transient = 0) and each landscape feature variable to investigate potential differences in selection between resident and transient coyotes. Second, we subsetted resident and transient locations and constructed separate models to derive 2^nd^- and 3^rd^-order selection coefficients for each landscape feature without interactions. We included all landscape features described above in our global models sets because correlation between individual predictor variables was low or modest (all *r* < 48%).We conducted model validation of the best model using *k*-fold cross-validation and then tested for predictive performance using area under the curve (AUC) [46–49]. This cross-validation is based on partitioning the data into *k* bins and performing *k* iterations of training and validation in which a different bin of the data is held out for validation, while remaining *k*–1 bins are used for the training set. We used 10 folds (*k* = 10) to estimate performance of RSF models. Area under the curve of a receiver operating characteristic (ROC) curve represents the relative proportions of correctly and incorrectly classified predictions over a range of threshold levels by plotting true positives versus false positives for a binary classifier system.

## Results

During 2009–2011, we fit 28 coyotes with GPS radio collars for monitoring. During this period, the Red Wolf Recovery Program also radio monitored 12 sterile coyote pairs (comprising about 20 radio-collared coyotes) each year. Each year, approximately 20 radio-collared coyotes were not associated with known packs or breeding pairs and were assumed to be transients. Monitoring data collected after release indicated 14 coyotes were residents and 14 were transients. Eight (57%) transient coyotes eventually established residency during the study. Mean (±SE) mass and age of coyotes monitored were 14.0 kg ± 0.4 and 2.5 yrs ± 0.2, respectively. Mass (*t*_26_ = 2.75, *P* = 0.010) and age (*t*_26_ = 2.23, *P* = 0.034) of resident coyotes were greater than transients (Table 1). Additionally, body measurements of coyotes sampled for this study were consistent with body measurements reported in Hinton and Chamberlain [50]. Mean resident home-range size (*t*_45_ = 0.03, *P* = 0.981) and resident core area (*t*_45_ = 0.26, *P* = 0.797) of coyotes did not differ between seasons (Table 1); resident home-range sizes ranged from 13.4 km² to 47.3 km². Although we detected no seasonal differences in the size of transient biding areas (*t*_17_ = 1.07, *P* = 0.296), our data suggest transient ranges were greater during the harvest season of agricultural crops (Table 1; *t*_17_ = 1.86, *P* = 0.080). Transient-range sizes ranged from 64.5 km² to 633.4 km².

**Table 1:** Mean (± SE) body mass, age, and space use of resident and transient coyotes in northeastern North Carolina during 2009–2011.

			Size of area used (km²)					
			Growing^1^		Harvest^2^		Composite^3^	
Coyote status	Mean mass (kg)	Mean age (yr)	95%^4^	50%^5^	95%	50%	95%	50%
**Resident**	14.7 ± 0.4	2.7 ± 0.2	24.1 ± 2.3	4.0 ± 0.5	25.0 ± 2.8	4.0 ± 0.4	27.2 ± 2.0	4.2 ± 0.4
**Transient**	12.3 ± 0.7	1.6 ± 0.5	212.5 ± 58.0	11.6 ± 4.1	296.9 ± 55.0	21.7 ± 3.9	307.9 ± 44.9	20.6 ± 3.2

^1^Growing season space use was defined as areas used during March through August.

^2^Harvest season space use was defined as areas used during September through February.

^3^Composite space use was defined as the total area used.

^4^95% probability contour calculated from dynamic Brownian bridge movement models used to estimate the sizes of resident home ranges and transient ranges.

^5^50% probability contour calculated from dynamic Brownian bridge movement models used to estimate the sizes of resident core areas and transient biding areas.

**Fig 2:**
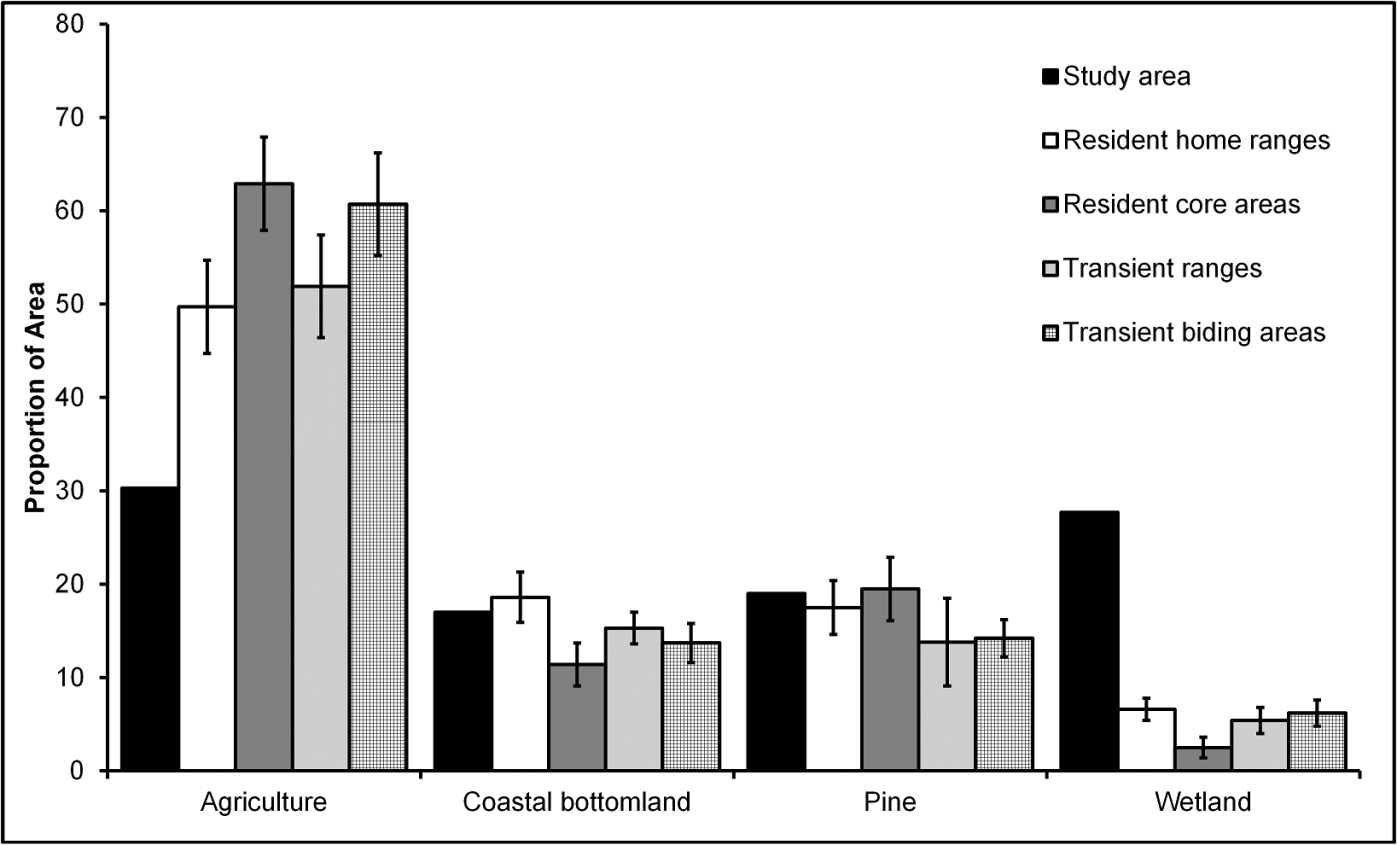
Habitat availability and habitat proportions of space used by resident and transient coyotes in northeastern North Carolina during 2009–2011. Asterisks above the bars represent statistical differences among areas within habitat classes (*P* < 0.05, Tukey’s test). Study area proportions are shown for reference.

**Fig 3:**
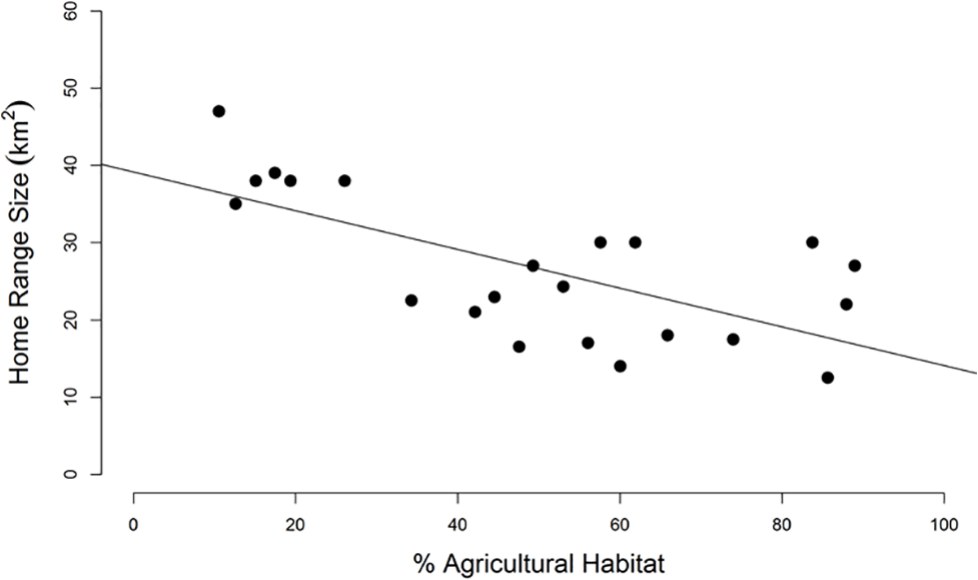
Home-range sizes of resident coyotes regressed against the percentages of agricultural habitats within home ranges (*r* ^2^ = 0.39, *P* < 0.001).

Resident home ranges, resident core areas, transient ranges, and transient biding areas of coyotes comprised mostly agriculture, coastal bottomland forest, and pine forest (Fig 2). Home-range sizes of residents were negatively correlated with the percentage of agricultural habitats found within home ranges (*r* ^2^ = 0.32, *P* = 0.003; Fig 3). We detected no difference in the proportion of habitat that comprised these 4 area measurements (resident home ranges, resident core areas, transient ranges, transient biding areas) for agriculture (*F*_3, 72_ = 1.66, *P* = 0.184), coastal bottomland forest (*F*_3, 72_ = 1.87, *P* = 0.142), and pine forest (*F*_3, 72_ = 0.81, *P* = 0.490; (Fig 2). Core areas used by resident coyotes contained proportionally less wetland than home ranges, transient ranges, and biding areas (*F*_3, 72_ = 5.51, *P* = 0.002).

We used distance to 6 landscape features (agriculture, coastal bottomland forest, pine forest, wetland, edge, and roads) to develop RSFs and map relative probability of habitat use by transient and resident coyotes separately. Model fit improved substantially by accounting for residency status and fitting interactions between resource variables and residency status to explicitly test for differences in habitat selection between residents and transients, providing support that coyote status affects resource selection (Tables 2 and 3). We created 4 subset models that included 2^nd^- and 3^rd^-order selection for resident and transient coyotes (Table 4). With the exception of pine forest, all other covariates were important predictors of transient occurrence at the landscape level in which transients selected agriculture and roads, and avoided coastal bottomland forest, wetlands, and edges (2^nd^-order selection; Table 5). Agriculture and roads were the only important predictors for transient 3^rd^-order selection (Table 6). All covariates were important predictors of resident habitat selection at the landscape level (2^nd^-order selection; Table 5). Agriculture, coastal bottomland forest, wetlands, edge, and roads were important predictors at the home-range level (3^rd^-order selection; Table 6). Although residents selected for all landscape features except pine forests at the landscape level, residents selected pine forests and avoided wetlands and roads at the home-range level (Tables 5 and 6). Our *k*-fold cross-validation correctly classified 88% of the resident locations for the selection model comparing resident and transient locations. Similarly, *k*-fold cross-validation correctly classified 80% and 76% of the locations for the best 2^nd^- and 3^rd^-order selection models for resident coyotes, respectively, whereas 77% of the locations for the best 2^nd^- and 3^rd^-order selection models for transient coyotes were correctly classified. Model performances of the best models for transient and resident coyotes ranged from poor to fair. The area under the curve value for the selection model comparing residents to transients was 78%. Area under the curve values were 73% and 63% for 2^nd^- and 3^rd^-order selection models of residents, respectively. Area under the curve values were 69% and 61% for 2^nd^- and 3^rd^-order selection models of transients, respectively.

**Table 2:** Comparison of model fit among the null model, and models with and without interactions used to test hypotheses about coyote resource selection at 2^nd^ and 3^rd^ order in northeastern North Carolina, 2009–2011. Shown are Akaike’s Information Criteria for small sample sizes (AIC_c_), differences among AIC_c_ (ΔAIC), and the conclusion regarding whether there was strong support for the interaction.

Order of selection	Models	*k*	AIC_c_	Deviance	ΔAIC	Conclusions
**2** ^**nd**^	Interactions (Resident x each variable)	14	90,512	90,464	0.00	Interactions strongly supported
	No interactions	8	93,910	93,889	3,398	
	Null	2	105,753	105,749	15,241	
**3** ^**rd**^	Interactions (Resident x each variable)	14	101,970	101,922	0.00	Interactions strongly supported
	No interactions	8	103,088	103,067	1,118	
	Null	2	105,178	105,174	3,208	

**Table 3:** Summary of results from generalized linear mixed models with for 2^nd^- and 3^rd^-order resource selection models for coyotes in northeastern North Carolina during 2009–2011. Shown are β coefficients, standard error (SE), 95% confidence intervals (CI), *z*-scores, and *P*-values.

Order of Selection	Model variables	β	SE	95% CI	*z*	*P*
**2** ^**nd**^ **-Order**	Intercept	-0.430	0.053	-0.532, -0.327	-8.19	<0.001
	Agriculture	-0.522	0.050	-0.620, -0.425	-10.50	<0.001
	Coastal bottomland forest	0.096	0.022	0.054 0.139	4.46	<0.001
	Pine	0.042	0.024	-0.006, 0.089	1.73	0.083
	Wetland	0.098	0.021	0.056, 0.140	4.56	<0.001
	Edge	0.220	0.046	0.130, 0.310	4.78	<0.001
	Road	-0.599	0.027	-0.652, -0.545	-21.88	<0.001
	Agriculture x Resident	-2.339	0.083	-2.502, -2.176	-28.11	<0.001
	Coastal bottomland forest x Resident	-0.533	0.028	-0.588, -0.478	-18.96	<0.001
	Pine x Resident	0.440	0.032	0.378, 0.502	13.97	<0.001
	Wetland x Resident	0.203	0.028	0.149, 0.258	7.23	<0.001
	Edge x Resident	-0.349	0.067	-0.481, -0.218	-5.21	<0.001
	Road x Resident	0.207	0.034	0.141, 0.273	6.15	<0.001
**3** ^**rd**^ **-Order**	Intercept	-0.051	0.070	-0.188, 0.085	-0.736	0.462
	Agriculture	-0.250	0.026	-0.301, -0.199	-9.638	<0.001
	Coastal bottomland forest	-0.032	0.019	-0.070, 0.006	-1.668	0.0954
	Pine	-0.044	0.019	-0.081, -0.007	-2.302	0.021
	Wetland	0.025	0.020	-0.014, 0.064	1.269	0.204
	Edge	-0.032	0.025	-0.080, 0.017	1.280	0.201
	Road	-0.168	0.015	-0.198, -0.138	-11.02	<0.001
	Agriculture x Resident	-0.936	0.047	-1.028, -0.844	-19.93	<0.001
	Coastal bottomland forest x Resident	-0.130	0.026	0.001, 0.001	5.78	<0.001
	Pine x Resident	-0.038	0.024	-0.010, 0.086	1.55	0.122
	Wetland x Resident	0.063	0.027	0.010, 0.116	2.34	0.020
	Edge x Resident	-0.049	0.042	-0.130, 0.032	-1.18	0.239
	Road x Resident	0.301	0.019	0.263, 0.338	15.53	<0.001

**Table 4:** Summary of generalized linear mixed models for predicting coyote habitat use in four groups corresponding to different hypotheses of landscape features potentially affecting 2^nd^- and 3^rd^-order habitat selection by transient and resident coyotes in northeastern North Carolina, 2009–2011. Shown are Akaike’s Information Criteria for small sample sizes (AIC_c_) and differences among AIC_c_ (ΔAIC).

Status	Order of selection	Model	*k*	AIC_c_	Deviance	ΔAIC
**Transient**	2^nd^	Full model	8	25,599	25,578	0
		No wetlands–AG^1^+CB^2^+PI^3^+ED^4^+RD^5^	7	25,614	25,596	14
		No forests–AG+WL^6^+ED+RD	6	25,615	25,601	16
		No agriculture–CB+PI+WL+ED+RD	7	25,704	25,690	108
		No linear features–AG+CB+PI+WL	6	26,239	26,224	639
**Resident**	2^nd^	Full model	8	64,822	64,806	0
		No wetlands–AG+CB+PI+ED+RD	7	65,106	65,088	279
		No linear features–AG+CB+PI+WL	6	65,253	65,237	427
		No forests–AG+WL+ED+RD	6	65,842	65,829	1016
		No agriculture–CB+PI+WL+ED+RD	7	66,917	66,899	2090
**Transient**	3^rd^	No wetlands–AG+CB+PI+ED+RD	7	24,052	24,034	0
		Full model	8	24,053	24,031	1
		No forests–AG+WL+ED+RD	6	24,060	24,045	8
		No agriculture–CB+PI+WL+ED+RD	7	24,143	24,126	91
		No linear features–AG+CB+PI+WL	6	24,150	24,135	98
**Resident**	3^rd^	Full model	8	75,693	75,671	0
		No wetlands–AG+CB+PI+ED+RD	7	75,712	75,694	19
		No forests–AG+WL+ED+RD	6	75,772	75,757	79
		No agriculture–CB+PI+WL+ED+RD	7	75,836	75,821	143
		No linear features–AG+CB+PI+WL	6	76,654	76,636	961

^1^ Agriculture

^2^ Coastal bottomland forest

^3^ Pine forest

^4^ Agriculture-forest edge

^5^ Roads

^6^ Wetlands

**Table 5:** Parameter estimates for 2^nd^-order resource selection functions for radio-collared coyotes in northeastern North Carolina during 2009–2011. Shown are β coefficients, standard error (SE), 95% confidence intervals (CI), *z*-scores, and *P*-values.

2^nd^-Order	Model variables	β	SE	95% CI	*z*	*P*
**Transient**	Intercept	-0.040	0.023	-0.090, 0.007	-1.71	0.088
	Agriculture	-0.522	0.050	-0.619, -0.425	-10.53	<0.001
	Coastal bottomland forest	0.091	0.022	0.049, 0.0133	4.25	<0.001
	Pine	0.041	0.024	-0.006, 0.088	-1.72	0.085
	Wetland	0.091	0.046	0.049, 0.132	4.26	<0.001
	Edge	0.221	0.046	0.131, 0.310	4.82	<0.001
	Road	-0.594	0.027	-0.648, -0.541	-21.95	<0.001
**Resident**	Intercept	-0.673	0.032	-0.742, -0.611	-20.81	<0.001
	Agriculture	-2.888	0.067	-3.020, -2.758	-43.21	<0.001
	Coastal bottomland forest	-0.437	0.018	-0.472, -0.402	-24.30	<0.001
	Pine	0.477	0.020	0.437, 0.517	23.43	<0.001
	Wetland	-0.299	0.018	0.228, 0.335	16.47	<0.001
	Edge	-0.131	0.049	-0.229, -0.036	-2.68	0.007
	Road	-0.390	0.020	-0.428, -0.351	-19.86	<0.001

**Table 6:** Parameter estimates for 3^rd^-order resource selection functions for radio-collared coyotes in northeastern North Carolina during 2009–2011. Shown are β coefficients, standard error (SE), 95% confidence intervals (CI), *z*-scores, and *P*-values.

3^rd^-Order	Model variables	β	SE	95% CI	*z*	*P*
**Transient**	Intercept	-0.477	0.750	-2.183, 1.091	-0.64	0.525
	Agriculture	-0.253	0.026	-0.304, -0.202	-9.64	<0.001
	Coastal bottomland forest	-0.034	0.021	-0.074, 0.007	-1.64	0.101
	Pine	-0.059	0.020	-0.097, -0.021	-3.01	0.003
	Wetland	0.030	0.021	-0.011, 0.072	1.44	0.151
	Edge	-0.031	0.025	-0.080, 0.018	-1.23	0.219
	Road	-0.159	0.016	-0.190, -0.129	-10.05	<0.001
**Resident**	Intercept	-0.707	0.293	-1.345, -0.124	-2.42	0.016
	Agriculture	-1.180	0.039	-1.257, -1.103	-30.07	<0.001
	Coastal bottomland forest	-0.161	0.018	-0.196, -0.125	-8.85	<0.001
	Pine	-0.016	0.015	-0.046, 0.014	-1.02	0.307
	Wetland	0.087	0.018	0.051, 0.123	4.73	<0.001
	Edge	-0.066	0.034	-0.131, 0.001	-1.96	0.050
	Road	0.139	0.012	0.115, 0.162	11.53	<0.001

**Fig 4:**
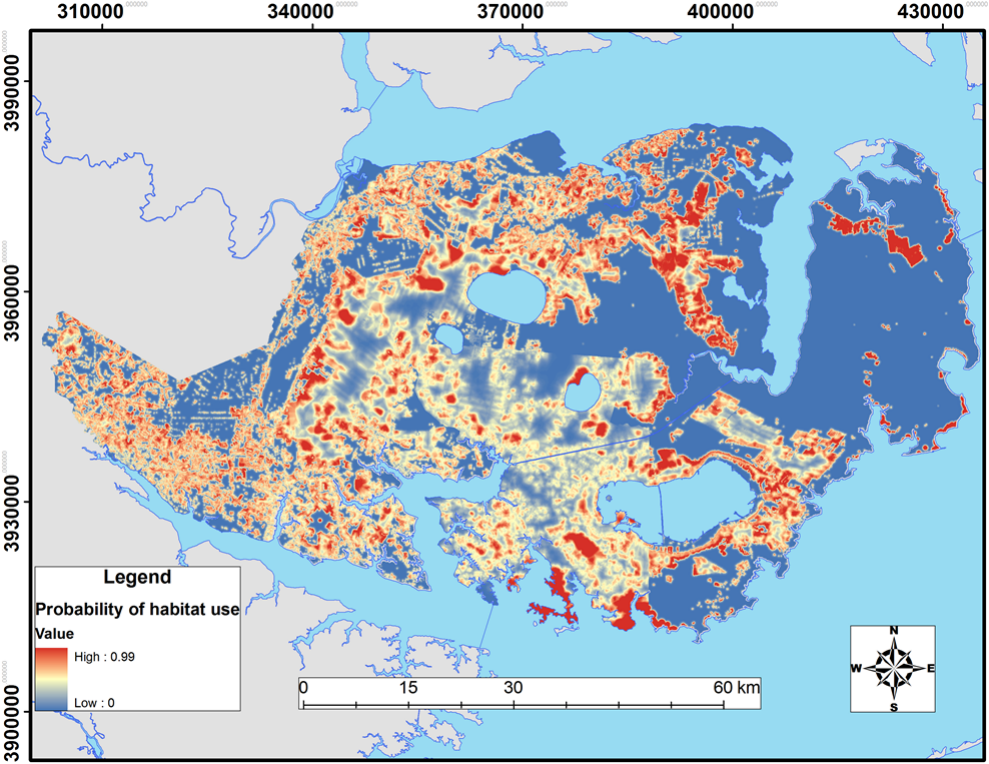
Relative probability of 3^rd^-order habitat selection by resident coyotes across the Albemarle Peninsula in northeastern North Carolina during 2009–2011.

**Fig 5:**
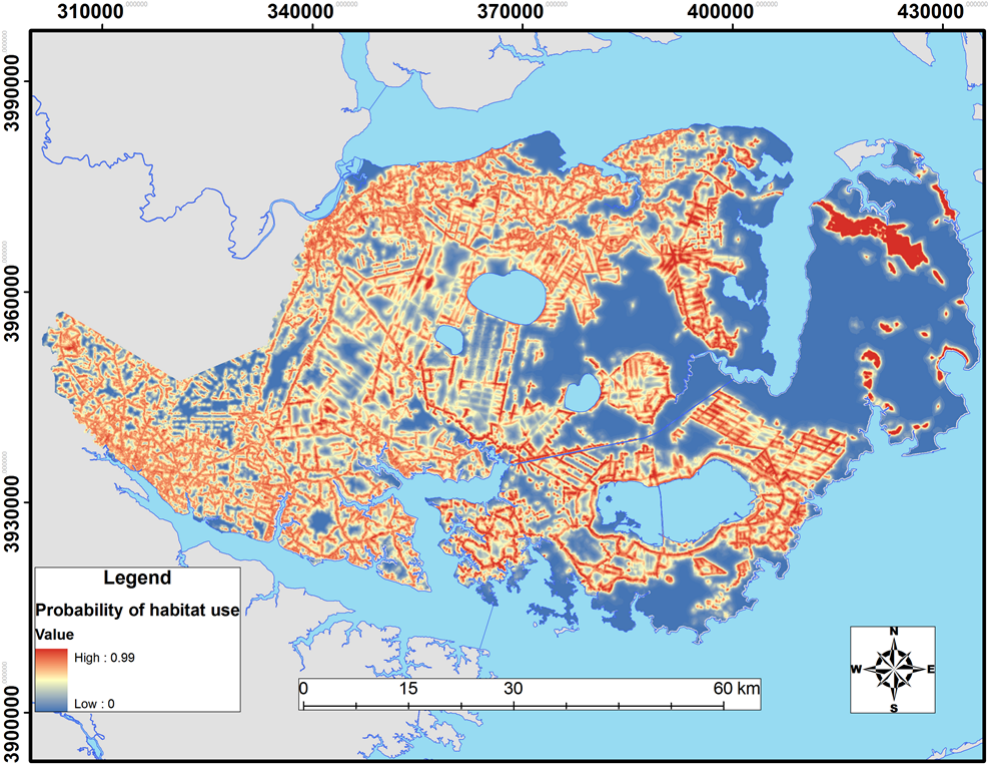
Relative probability of 3^rd^-order habitat selection by transient coyotes across the Albemarle Peninsula in northeastern North Carolina during 2009–2011.

Spatially, differences in habitat selection between residents and transients revealed substantial heterogeneity in the response to the agricultural-forest habitat matrix of the Albemarle Peninsula (Figs 4 and 5). Compared to transients, resident coyotes showed greater selection for agriculture, coastal bottomland forest, and edge and lower selection for roads (Tables 5 and 6).

## Discussion

**Fig 6:**
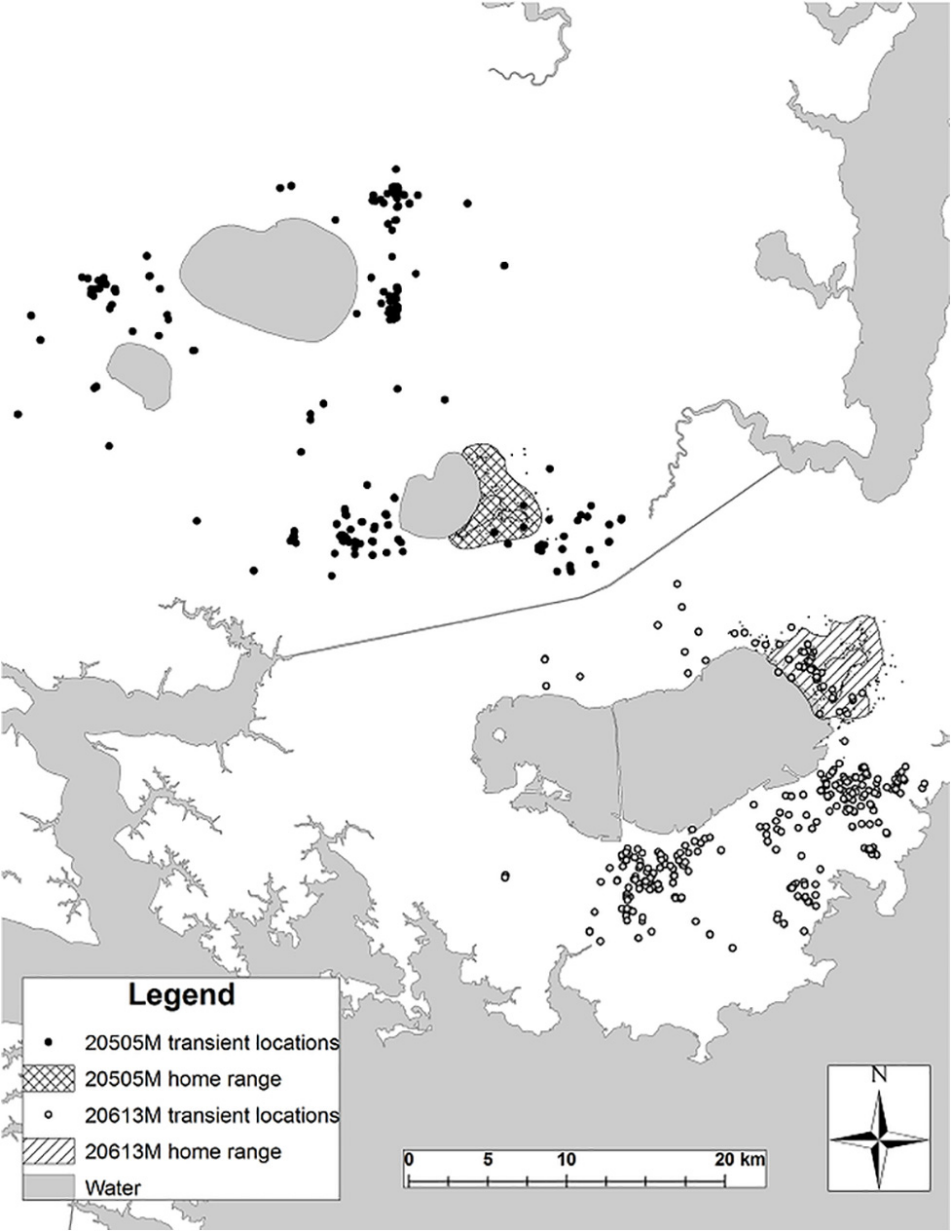
Transient locations and estimated home ranges of coyotes 505M and 613M in eastern North Carolina. Coyote 505M was monitored as a transient from 16 April 2009 until 31 May 2009. Coyote 505M established a territory approximately 1 June 2009 and maintained it until 27 October 2009 when he was displaced by a neighboring red wolf pack. Coyote 613M was monitored as a transient from 7 January 2011 until 4 April 2011. Coyote 613M established a territory approximately 5 April 2011 after the resident red wolf pack dissolved after the death of a breeder. Coyote 613M was monitored as a resident from 5 April 2011 until 16 August 2012 when his GPS collar failed.

Our findings indicate that transient individuals may play a crucial role in dynamic space-use patterns of coyotes. Similar to other studies [7,11,51], our results indicate that approximately 70% of coyotes in eastern North Carolina are likely residents whereas the remaining 30% are transients. Transients consisted of younger and smaller individuals than residents and this may indicate that most transients are dispersing juveniles. However, as breeding pairs and packs are disrupted via natural or anthropogenic sources, older individuals who previously were residents may become transient as well. For instance, after coyote 505M (Fig 6) established a home range, he was displaced by a neighboring red wolf pack and was a transient for approximately 15 weeks until establishing a second territory with a female red wolf. Under the direction of the Recovery Program, 505M was removed during October 2011 so the female red wolf would be available to potential red wolf mates. Indeed, approximately 4 weeks later, a male red wolf moved in and formed a breeding pair with the female red wolf (USFWS, *unpublished data*).

Throughout North America, coyote home-range sizes typically vary between 2.5 and 70 km^2^ and the home-range sizes we documented for eastern North Carolina are typical of those reported in other studies (see Table 22.4 in Bekoff and Gese [52] and Table 21 in Leopold and Chamberlain [53]). Home ranges of coyotes in our study ranged between 13 and 47 km^2^ and did not exceed 47 km^2^, indicating that coyotes may have an upper limit to the areas they can effectively exploit and defend as territories. Although regional variability in coyote home-range sizes can be attributed to adjustments of space use patterns to local environmental conditions, the minimum and maximum size of coyote home-ranges is likely driven by metabolic costs, which varies with body mass [54,55]. Coyotes can only defend a finite area while maintaining an optimal foraging strategy commensurate with the distribution and availability of prey in their territories [56,57]. Home ranges of resident coyotes were stable and did not vary between seasons, suggesting that coyotes may not adjust home-range size to immediate demand but rather potential demand. In other words, coyotes are likely aware of potential changes in the environment prior to establishing residency and acquire enough space to accommodate seasonally varying needs and resource availability.

Instability (i.e., shifting patterns) of space use and use of areas greater than 65 km^2^ were characteristic of transient coyotes. Despite their wide-ranging (64.5 km² to 633.4 km²) space-use patterns, many transients exhibited localized movements (i.e., clusters of locations) for several weeks that averaged about 21 km^2^ and those areas appeared analogous to home ranges in both size and habitat composition. We referred to them as biding areas [20] and 7 of 8 (88%) residents who were initially transients established home ranges in or nearby their biding areas (Fig 6). We suggest this behavior may provide benefits to coyote populations because it increases survivorship of transients via familiarity of areas they roam, allow transients to assess potential areas prior to establishing home ranges, and, when opportunities arise, replace residents upon death. However, this relationship requires further investigation. Territorial behavior in coyotes involves a strategy to increase reproductive success among residents holding space [58]. Although this prevents transients from reproducing, transiency is likely an important trait that allows populations to recover rapidly after suffering drastic and extensive mortality. This may be particularly important for coyote populations to persist where they are heavily exploited. For example, 7 coyotes monitored in this study replaced resident coyotes and red wolves that were killed during the study [59].

Relationships between agriculture and forest habitat and coyote space use in northeastern North Carolina are similar to those reported for studies in the Northeast and indicate general selection for open, treeless environments [60–62]. Coyotes typically centered territories on edges of agricultural fields and forests with higher percentages of agriculture in the interior (i.e., core areas) as forest habitat increased in outer fringes. During harvest season (autumn through winter), coyotes typically loafed in forest habitats within 50–300 m of edges adjacent to agricultural fields and roads. As winter wheat reached heights of approximately 0.5 m during the growing season (spring through summer), coyotes abandoned forest habitats to loaf in wheat fields when available and then shifted to corn later in the season as wheat was harvested (J. Hinton, *personal observation*). Home ranges were smaller where agriculture became the predominant habitat type (Fig 3), whereas the opposite pattern occurred for forested habitats. For example, the smallest home-range size (13.4 km^2^) was that of a female coyote, which contained approximately 56% agricultural and 30% forested habitat. Of her 1,987 GPS locations, approximately 87% occurred in agriculture. In contrast, the home range of a female coyote with the largest home-range size (47.3 km^2^) consisted of approximately 10% agricultural and 70% forested habitat. Of her 2,296 GPS locations, approximately 35% were in agriculture.

Although habitat compositions of space used by resident and transient coyotes were similar, patterns of habitat selection differed. Direct comparison between residents and transients revealed that both selected for agriculture but coastal bottomland forest and edges were selected more by residents whereas transients were more likely to show selection for roads. Consequently, resident coyotes tended to establish territories in predominantly agricultural areas whereas transient coyotes appeared to center their movements and biding areas proximate to these same habitats via road networks (Fig 5). Indeed, models of 3^rd^-order selection indicated only agriculture and roads were important for predicting transient habitat use.

Use of roads was a primary difference in habitat use between residents and transients at the 3^rd^-order selection level. Coyote populations are adept at exploiting anthropogenic landscape features [36,63], and we suggest the use of roads by transients may be related to 2 important aspects of transient ecology. First, roads may provide benefits to transient coyotes through efficient movements that improve foraging opportunities and reduce energetic costs related to shifting and expansive space use. The use of roads may also permit transients to move efficiently through unsuitable habitat (i.e., inundated forested habitats and wetlands). For example, coyote use of bridges to cross waterways has been observed [63]. Indeed, we documented several of the transient coyotes crossing bridges [20]. Second, most contact between transient and resident coyotes likely occur through passive and indirect interactions (i.e., scent marking). As observed in gray wolves (*Canis lupus*; [64,65]) and red wolves [66], roads and linear corridors may enhance line of sight and olfactory senses of *Canis* species and facilitate detection of conspecifics and their territorial boundaries. However, use of roads are known to expose coyotes to increase risks of mortality and how coyotes make trade-offs between costs and benefits associated with using roads will need to be further assessed [38].

The extent of study areas can make it difficult to understand the probability of occurrence of coyotes on the landscape. Although our probability maps of predicted habitat selection reveal distinct gradients of habitat suitability on the Albemarle Peninsula, our AUC scores were low. Low AUC values indicate the ability of the habitat models to discriminate between coyote and random locations was limited, but do not necessarily imply low model accuracy [67]. We believe our low AUC values do not imply low model accuracy because coyotes are generalists and AUC values for species with broad requirements tend to be low to denote their widespread distribution [66]. Second, models of 2^nd^-order selection had greater AUC values than 3^rd^-order models, indicating the effect that geographic extent can have on AUC values. In this case, random locations used in 2^nd^-order selection models were typically much further from areas of confirmed use (e.g., resident home ranges and transient ranges) than those used in 3^rd^-order selection models. Consequently, random locations in 2^nd^-order selection orders were more distinct in their characteristics than those in 3^rd^-order selection and were better predicted (i.e., greater model discrimination). In other words, by simply increasing the geographic extent to areas beyond those occupied by radio-collared coyotes we artificially increased our AUC values. Therefore, it is likely that we could not assess true accuracy of different models because 2^nd^- and 3^rd^-order selection models differed in the total extent analyzed [67].

Although transient coyotes are commonly perceived as subordinate individuals who are excluded to suboptimal space unoccupied by residents [5,7,8,15], our knowledge about the role of transients in coyote ecology remains limited. Because territories are also transitory and space is frequently gained and lost by individuals, coyotes, irrespective of age and social status, can become transient through a number of causes. When released from their territories, coyotes are capable of traversing over large areas because of their relatively large body size, physiology, and overall need to move in response to ecological demands. Therefore, behaviors associated with transiency involve important decisions by individuals that permit coyotes to seek out new territories and breeding opportunities broadly across the landscape. During our study, transient coyotes typically replaced lost mates of residents. When residents lost mates, we documented surviving residents permitting several transients of the opposite sex into their territories to select a new mate. Once a new mate was selected, the resident coyote regained exclusive control of the territory. Because of these observations, we assumed biding areas of transients may represent attempts of transients to establish territories through mate selection. As a result of dynamic space use patterns documented in our study, we believe transiency may be an important life history trait because it facilitates metapopulation dynamics through dispersal and replacement of resident breeders [68–70]. Coyotes have become an apex predator throughout eastern North America and our findings provide insights into the potential role of transients in coyote ecology.
